# Machine learning risk estimation and prediction of death in continuing care facilities using administrative data

**DOI:** 10.1038/s41598-023-43943-9

**Published:** 2023-10-18

**Authors:** Faezehsadat Shahidi, Elissa Rennert-May, Adam G. D’Souza, Alysha Crocker, Peter Faris, Jenine Leal

**Affiliations:** 1 Electrical and Software Engineering, University of Calgary, Calgary, AB Canada; 2 Community Health Sciences, University of Calgary, Calgary, AB Canada; 3 Department of Medicine, University of Calgary, Calgary, AB Canada; 4 Centre for Health Informatics, University of Calgary, Calgary, AB Canada; 5 Clinical Information Systems, Alberta Health Services, Calgary, AB Canada; 6 Analytics, Alberta Health Services, Calgary, AB Canada; 7 O’Brien Institute for Public Health, University of Calgary, Calgary, AB Canada; 8 Department of Microbiology, Immunology, and Infectious Diseases, University of Calgary, Calgary, AB Canada; 9 Snyder Institute for Chronic Diseases, University of Calgary, Calgary, AB Canada; 10 AMR – One Health Consortium, University of Calgary, Calgary, AB Canada; 11 Infection Prevention and Control, Alberta Health Services, Calgary, AB Canada

**Keywords:** Risk factors, Engineering, Biomedical engineering, Health care

## Abstract

In this study, we aimed to identify the factors that were associated with mortality among continuing care residents in Alberta, during the coronavirus disease 2019 (COVID-19) pandemic. We achieved this by leveraging and linking various administrative datasets together. Then, we examined pre-processing methods in terms of prediction performance. Finally, we developed several machine learning models and compared the results of these models in terms of performance. We conducted a retrospective cohort study of all continuing care residents in Alberta, Canada, from March 1, 2020, to March 31, 2021. We used a univariable and a multivariable logistic regression (LR) model to identify predictive factors of 60-day all-cause mortality by estimating odds ratios (ORs) with a 95% confidence interval. To determine the best sensitivity–specificity cut-off point, the Youden index was employed. We developed several machine learning models to determine the best model regarding performance. In this cohort study, increased age, male sex, symptoms, previous admissions, and some specific comorbidities were associated with increased mortality. Machine learning and pre-processing approaches offer a potentially valuable method for improving risk prediction for mortality, but more work is needed to show improvement beyond standard risk factors.

## Introduction

The COVID-19 pandemic had significant scientific, medical, and social effects on the population^1–3^. However, COVID-19 does not equally affect all members of society^4–6^. Long-term care (LTC) residents (frail older adults who are not able to live by themselves and require medical care^4,5,7–9^) and designated supportive living (DSL) residents (older adults who can live independently but with support^10^) initially became the central point of the COVID-19 pandemic globally and Canada was not an exception^5,7^, as more than half of the deaths related to COVID-19 occurred in LTC facilities^7–9,11,12^. As LTC and DSL residents are at high risk of mortality from COVID-19, it is important to understand the predictors that are highly associated with adverse outcomes in this population.

One potential option to explore predictors of adverse outcomes includes standard logistic regression^15^. However, some studies have explored predictors using machine learning (ML) models over LR due to their superior predictive performance, especially for large-sized administrative healthcare data^13–15^. There are limitations for the LR models, including handling continuous variables, restricting the number of entered variables, and managing highly correlated variables^16^. Also, in a comprehensive survey study, it was found that the higher the degree of the class imbalance in the data, the greater the effects of the class imbalances on model performance^17^. Researchers have explored the likelihood of future outcomes using ML techniques, such as artificial neural networks (ANN)^15^, support vector machines (SVM)^18^, extreme gradient boosting (XGBoost)^19^, or random forests (RF)^13^ and advocated that ML models had better predictive performances than the standard LR model^14^.

The objective of our work is to compare LR and ML models to identify predictors of all-cause mortality^20^ among LTC and DSL residents in Alberta, Canada following a negative or positive COVID-19 test. Additionally, we developed and evaluated ML models, comparing them with LR models using the area under the curve (AUC) based on the Receiver Operating Characteristic (ROC) curve and sensitivity. This analysis aims to provide insights into all-cause mortality determinants and assess ML potential in improving predictive performance and patient outcomes in healthcare settings.

## Methods

### Study design and data access

This study was a retrospective cohort study using secondary data. The manuscript followed the observational routinely collected data (RECORD) reporting guideline^21^. The study adherence to the RECORD checklist items was demonstrated in Appendix 1. The study was reviewed and approved by the University of Calgary Ethics REB20-0688. Data Disclosure Agreements were developed with Alberta Health Services (AHS).

### Setting and years of data

The data were collected to develop a population-based COVID-19 analytics and research database to understand local epidemiology and resource use implications of COVID-19. The data included every person in Alberta who tested for COVID-19 from March 1, 2020, to March 31, 2021. However, only the first positive record and the first negative record (for those people that never test positive) per person were included.

### Data sources

The laboratory data, including age, sex, specimen collection information (location, and date), and the interpreted result (being symptomatic) were retrieved from the Alberta health services enterprise data warehouse (EDW)^22,23^. The specimen collection location based on date and the residency information were accessed through Alberta continuing care information system (ACCIS)^24,25^. Outpatient data, including previous ambulatory visits, previous procedures, and specimen collection locations based on data were accessed from the national ambulatory care reporting system (NACRS)^26,27^. Chronic conditions^28^ came from practitioner claims under the Alberta health care insurance plan (AHCIP)^29^ and discharge abstract database (DAD) databases. Also, previous hospitalizations, previous procedures, previous special care unit (SCU) visits, and specimen collection locations based on data were acquired from the DAD database^30^. The mortality data was gained from the Alberta vital statistics (AVS) database^31^. All of the administrative data codes^32^ used in this study are included in Appendix 2.

### Data linkage and data preparation

The Data were linked using PHN (personal health number) or ULIs (universal lifetime identifier). Patient identifiers, including PHN, ULI, names, date of birth, and postal codes were all removed (Scrambled ULIs were provided). Afterward, all dates were removed and counts of days between the index date (COVID-19 collection date), and the date of interest were calculated, according to AHS de-identification policies. The flowchart diagram depicting the cohort creation, data cleaning, and analyses was included in Appendix 3.

### Cohort study of LTC/DSL residents

The overall tested individuals within the data set included 1.8 million records. Among all tested populations, our study cohort comprised 25,586 individuals who were residents of LTC or DSL facilities in Alberta and had confirmed first positive or first negative (for those who were never positive) COVID-19 test results between March 1, 2020, and March 31, 2021.

### Variables of interest

In alignment with a previous study^33^, comorbidities were assessed based on historical data from the two years preceding the index date. We also completed a one-year lookback before episodes of infection for the number of admissions, procedures, and special care unit (SCU) visits to establish a baseline of healthcare utilization^33^.

The primary outcome in our study was all-cause mortality within 60 days of a resident’s first COVID-19 polymerase chain reaction (PCR) positive or negative test result. According to an early study^34^, there is a time lag of two to eight weeks (60 days) between COVID-19 cases (index date) and death. In our study, we utilized all-cause mortality^20^ data within 60 days of a positive or negative COVID-19 test, as this information was available in vital statistics.

The selected covariates were based on both clinical expertise and previous literature that has examined the association between age and the Elixhauser comorbidity index^35^, as well as prior admissions^36^ and their connection to severe COVID outcomes. So, the selected covariates encompassed various factors, including age, demographic characteristics (sex, LTC vs DSL resident, and specimen collection location), comorbidities (based on the Elixhauser index), the number of previous procedures (inpatient and outpatient), the number of previous admissions (hospital and ICU), symptomatic status, specimen year-month collection, and the results of the PCR test.

### Statistical analysis

Missing values were addressed by employing a strategy that involves imputation from other relevant columns, illustrated in the flowchart in Appendix 3. To illustrate, when dealing with missing values in the "Symptomatic during collection" feature, we have imputed these values using the corresponding test results. Additionally, we addressed outliers within the dataset, removing a minimal percentage of records (specifically, one record from a 17-year-old individual and five with unidentified sex), which collectively constituted only  0.02% of all records. The removal of the 17-year-old individual from the study cohort was carried out to ensure that the data accurately represents the target population, which includes individuals older than 18 years of age. The exclusion of individuals of unidentified sex was performed to address potential errors in data collection or recording and to enhance the overall quality and accuracy of the dataset. The steps were illustrated in the flowchart in Appendix 3.

In this study, descriptive statistics were utilized to provide a comprehensive overview of the cohort characteristics. Descriptive statistics including frequencies and percentages for categorical variables and means with standard deviations (SD) for normally distributed continuous variables and medians with interquartile ranges (IQR) for skewed continuous variables were used to describe the characteristics of the cohort.

In this study, we aimed to directly estimate the odds^37^ of mortality occurring. Given a binary outcome variable, we chose standard logistic regression (LR)^38^ over modified Poisson regression as LR is more suitable for estimating odds in binary outcomes. Univariable LR was used to identify individual predictive factors of 60-day mortality by estimating odds ratios (ORs)^39^ with a 95% of confidence interval (CI). A multivariable LR was applied to examine the joint association of all risk factors with 60-day mortality with adjusted ORs (aORs) and 95% CIs.

For cross validating the predictive models, the data were split into a training set (90% of the sample data) and a test set (10% of the sample) randomly^40^. The forward selection method was used to add the variables to the predictive models by iteratively adding features (predictor variables) to the model^41^. We used sensitivity as a measure to check which predictors are to be included in the model.

While useful for evaluating standard LR models, the AUC, sensitivity, and specificity values do not explicitly identify the best cut points^42^. To identify ideal cut points Youden index (J) method was proposed^43^. Youden’s index measures the difference between the true positive rate and the false positive rate across all potential cut-point values to calculate the perfect cut-point^44,45^.

In this study, the pre-processing techniques include random over-sampling to balance the classes and power transformation (PT) to normalize the data^17,46–48^. We employed power transformation to address non-normality and skewness in the data distribution and successfully normalized the data^49^. This transformation was found to be more effective in terms of performance, leading to improved results in our analysis.

Oversampling techniques were proven to outperform under-sampling methods by addressing data imbalance without losing valuable information, ultimately leading to improved model performance^50^. So, the random over-sampling technique (ROTE) and synthetic minority over-sampling technique (SMOTE)^51,52^ were applied separately for the training set. The test set was kept untouched for the final performance report and the LR models using initial data using 0.5 threshold (model 1), initial data using 0.083 threshold (model 2), SMOTE using 0.46 threshold (model 3), and ROTE using 0.42 threshold (model 4) were evaluated.

### Model development

Along with models 1–4 (LR models with pre-processing done), ML models, including RF, SVM, XGBoost, and ANN have been developed and tested to predict the 60-day incidence of mortality^13–15,18^. An alpha level of 0.05 (two-tailed) was used to assess statistical significance. We used Advanced Research Computing (ARC) cluster at the University of Calgary^53^, to submit several jobs in parallel. Each job had a unique input to do hyper-parameter optimization. In this examination, about 200,000 experiments were performed to detect the best hyper-parameters for all the ML models. The best results received by these hyper-parameters are illustrated in Appendix 4. These models were examined using normalized data and balanced classes. Data analysis and model development were performed by using Python programming language (version 3.9) and packages were illustrated in Appendix 5. As a popular scripting language for coordinating shell tasks, the bourne-again shell (Bash) was utilized to manage the resources in ARC^54^.

Several models were examined in this study. First was the RF model^55^, an ML algorithm, that works based on the majority decisions of several ‘decision trees’ that are centered on randomly selected variables in a dataset^56^. Second, SVM classifies by using a multidimensional hyperplane (to maximize the margin between the clusters) and a nonlinear function (kernel)^57^. Third, XGBoost was appealing since, when compared to the other classifiers, its default option had a great average performance^58^. XGBoost is based on a decision-tree ensemble algorithm that uses a gradient-boosting framework. Finally, ANN is becoming a common ML model in the field of health care^59–64^. This model has at least three layers, including input (receives the features), hidden (extracts the patterns based on weights), and output (presents the output)^59^. Each layer has nodes or neurons that are connected to their adjacent unit by a set of adjustable weights^62^. Like LR, the ANN models benefit from activation functions (like sigmoid) to produce the output^65^.

## Results

Among all LTC residents who were tested for COVID-19 from March 1, 2020, to March 31, 2021, six individuals were removed due to missing values in sex and outliers in age. Therefore, we had 25,580 continuing care residents (16,219 women [63.41%]; median age, 84 years [interquartile range, 80 + years]; of which 15,066 were LTC residents [58.9%], and 10,514 were DSL residents [41.1%]. Among all residents, 14,470 were tested in LTC [56.57%], 8748 were tested in DSL [38.11%], 743 were tested in ED [2.9%], and 619 were tested in hospital [2.42%]. Nearly 14% (n = 3489, 13.64%) had missing symptom information, 75.32% were asymptomatic, and 11.04% had reported symptoms at the time of testing. In this cohort, 3945 tested positive [15.42%] for COVID-19, and 93.19% (n = 23,838) had at least one comorbidity (Appendix 6).

Overall, 2590 residents (10.12%) died (1454 women [56.14%]; median age, 88 years [interquartile range, 80 + years]; of which 1893 were LTC residents [73.09%], and 697 were DSL residents [26.91%]. The largest proportion of deaths (18.38%) occurred among continuing-care residents tested for COVID-19 in April 2020. All characteristics are described in Table 1. The horizontal bar charts in Appendix 7 present the characteristics of continuing-care residents in Alberta who died. For 2,560 residents who died within 60 days, the number of previous mean hospital admissions for those who were tested positive for COVID was 0.7 compared to those who were tested negative for COVID which was 0.5. SCU admissions were 0.02 and 0.01 for those with and without COVID. For those who tested positive for COVID-19, the mean number of previous inpatient and outpatient procedures was 0.4 and 5.6, respectively. Those who were tested negative for COVID-19 also had a mean of 5.2 outpatient procedures and 0.3 inpatient procedures in the past.

**Table 1:** Characteristics of continuing care residents in Alberta who were confirmed tested positive or negative with a Covid-19 infection between March 1, 2020, to March 31, 2021.

Characteristic	60-day mortality		Total = 25,580
	Yes = 2590 (10.12%)	No = 22,990 (89.87%)	
Age category			
80 +	2022 (78.07)	14,646 (63.71)	16,668 (65.16)
70–79	386 (14.9)	4555 (19.81)	4941 (19.32)
60–69	144 (5.56)	2291 (9.97)	2435 (9.52)
50–59	28 (1.08)	994 (4.32)	1022 (4)
40–49	4 (0.15)	303 (1.32)	307(1.2)
30–39	4 (0.15)	156 (0.68)	160 (0.63)
18–29	2 (0.08)	45 (0.2)	47 (0.18)
Gender			
Female	1454 (56.14)	14,765 (64.22)	16,219 (63.41)
Male	1136 (43.86)	8225 (35.78)	9361 (36.59)
Specimen collection location			
LTC	1706 (65.87)	12,764 (55.52)	14,470 (56.57)
DSL	520 (20.08)	9228 (40.14)	9748 (38.11)
ED	217 (8.38)	526 (2.29)	743 (2.9)
H	147 (5.68)	472 (2.05)	619 (2.42)
Resident during collection			
LTC	1893 (73.09)	13,173 (57.3)	15,066 (58.9)
DSL	697 (26.91)	9817 (42.7)	10,514 (41.1)
Symptomatic during collection			
No	1573 (60.73)	17,695 (76.97)	19,268 (75.32)
Unknown	532 (20.54)	2957 (12.86)	3489 (13.64)
Yes	485 (18.73)	2338 (10.17)	2823 (11.04)
Result of the covid test			
Negative	1594 (61.54)	20,041 (87.17)	21,635 (84.58)
Positive	996 (38.46)	2949 (12.83)	3945 (15.42)
Specimen year-month collection			
2020-3 (original variant)	192 (7.41)	835 (3.63)	1027 (4.01)
2020-4 (alpha)	476 (18.38)	3226 (14.03)	3702 (14.47)
2020-5	271(10.46)	2054 (8.93)	2325 (9.09)
2020-6	338 (13.05)	9192 (39.98)	9530 (37.26)
2020-7	117 (4.52)	894 (3.89)	1011 (3.95)
2020-8	50 (1.93)	549 (2.39)	599 (2.34)
2020-9	59 (2.28)	514 (2.24)	573 (2.24)
2020-10 (delta)	198 (7.64)	1226 (5.33)	1424 (5.57)
2020-11	276 (10.66)	1389 (6.04)	1665 (6.51)
2020-12 (beta)	373 (14.4)	1827 (7.95)	2200 (8.6)
2021-1 (gamma)	199 (7.68)	846 (3.68)	1045 (4.09)
2021-2 (theta)	30 (1.16)	277 (1.2)	307 (1.2)
2021-3	11 (0.42)	161 (0.7)	172 (0.67)
Comorbidities			
Hypertension, uncomplicated	1307 (50.46)	11,751 (51.11)	13,058 (51.05)
Depression	1121 (43.28)	10,922 (47.51)	12,043(47.08)
Other neurological disorders	1060 (40.93)	9413 (40.94)	10,473 (40.94)
Diabetes	802 (30.97)	6557 (28.52)	7359 (28.77)

*LTC* long-term care, *DSL* designated support living, *ED* emergency department, *H* hospital.

The horizontal bar charts in Appendix 8 illustrate the multivariable associations between clinical risk factors and 60-day all-cause mortality in continuing-care residents in Alberta. In our univariable and multivariable analyses between resident characteristics and 60-day mortality, the following associations were observed (Table 2).

1. Sociodemographic characteristics: In both analyses, older age was associated with a higher probability of 60-day mortality. The odds of death increased by a factor of 1.05 per one-year increase in age [95% CI 1.04–1.05] (p < 0.01). Concerning other demographic risk factors, men had a higher risk than women for 60-day mortality (aORs 1.57 [95% CI 1.42–1.73]; p < 0.01). The risk of death was lower among DSL residents compared with LTC residents (DSL residents: aORs 0.54 [95% CI 0.41, 0.71], p < 0.01). As for LTC residents, 39% were male, 18% tested positive for COVID-19, 64% were 80 years old or older, and 92% had at least one comorbidity. For DSL residents, 33% were male, 13% tested positive, 66% were 80 years old or older, and 95% had at least one comorbidity. By comparison, the percentages of males and individuals who tested positive living in LTC were greater than those in DSL. For the locations where the specimen was collected, the highest risk of death was for the ones who were tested in the emergency department (aORs 4.54 [95% CI 3.57–5.77], p < 0.01); followed by those in the hospital (aORs 3.6 [95% CI 2.76–4.68], p < 0.01). There was no evidence that those residents tested while still residing in their DSL facility had decreased odds of death (aORs 0.97 [95% CI 0.72–1.30], p = 0.82]).
2. Clinical characteristics: In both analyses, having symptoms was associated with the highest probability of 60-day mortality. Compared with the residents with no symptoms, the adjusted odds of death were 2.02 (95% CI 1.77–2.32) times higher for the ones with symptoms, and 1.45 (95% CI 1.27–1.65) times higher for the residents with unknown symptoms. Additionally, those who tested positive for COVID-19 had a greater risk of 60-day mortality than those who tested negative for this disease (aORs 3.61 [95% CI 3.14–4.15]).
3. Hospital admission-related variables: Despite not reaching strict statistical significance, a discernible trend suggests that an increased number of previous hospital admissions (aORs 1.02 [95% CI 0.97–1.08], p = 0.44), SCU admissions (aORs 1.15 [95% CI 0.83–1.60], p = 0.41), and inpatient procedures (aORs 1.01 [95% CI 0.97–1.06], p = 0.59) were associated with higher odds of 60-day mortality.
4. Comorbidities: Among all residents who died, 1307 (50.46%) had uncomplicated hypertension, 1121 (43.28%) had depression, 1060 (40.93%) had neurological disorders, 809 (31.24%) had fluid and electrolyte disorders, and 802 (30.97%) had diabetes (Table 1). The most prevalent comorbidities were listed in Appendix 6. When considered together with clinical, inpatient, and demographic characteristics, among the most prevalent comorbidities we identified that fluid and electrolyte disorders (aORs 1.19 [95% CI 1.07–1.34]), and diabetes (aORs 1.11 [95% CI 1.00–1.23]) were associated with a higher probability of 60-day mortality. Metastatic cancer (aORs 1.58 [95% CI 1.14–2.19]) and chronic liver disease (aORs 1.51 [95% CI 1.15–1.99]) were the strongest risk factors of 60-day mortality (Table 2). The OR and aORs of the most prevalent comorbidities were listed in Appendix 9.

**Table 2:** Associations between clinical risk factors and 60-day cause-specific mortality in continuing care residents in Alberta who were confirmed tested first positive or first negative with a Covid-19 infection between, March 1, 2020, to March 31, 2021.

Characteristic	Univariable		Multivariable	
	ORs (95% CI)	p-value	AORs (95% CI)	p-value
Age	1.04 (1.03, 1.04)	< 0.01	1.05 (1.04, 1.05)	< 0.01
Gender				
Male	1.40 (1.29, 1.52)	< 0.01	1.57 (1.42, 1.73)	< 0.01
Female	1 [Reference]		1 [Reference]	
Specimen collection location				
LTC	1 [Reference]		1 [Reference]	
DSL	0.42 (0.38, 0.47)	< 0.01	0.97 (0.72, 1.30)	0.82
ED	3.09 (2.61, 3.64)	< 0.01	4.54 (3.57, 5.77)	< 0.01
H	2.33 (1.92, 2.82)	< 0.01	3.6 (2.76, 4.68)	< 0.01
Resident during collection				
LTC	1 [Reference]		1 [Reference]	
DSL	0.49 (0.45,0.54)	< 0.01	0.54 (0.41, 0.71)	< 0.01
Symptomatic during collection				
No	1 [Reference]		1 [Reference]	
Yes	2.33 (2.09, 2.61)	< 0.01	2.02 (1.77, 2.32)	< 0.01
Unknown	2.02 (1.82, 2.25)	< 0.01	1.45 (1.27, 1.65)	< 0.01
Result of the covid test				
Negative	1 [Reference]		1 [Reference]	
Positive	4.25 (3.89,4.64)	< 0.01	3.61 (3.14, 4.15)	< 0.01
Specimen year-month collection				
2020-3 (original)	6.25 (5.17, 7.57)	< 0.01	4.62 (3.71, 5.75)	< 0.01
2020-4 (alpha)	4.01 (3.47, 4.64)	< 0.01	2.96 (2.52, 3.48)	< 0.01
2020-5	3.59 (3.04, 4.24)	< 0.01	2.08 (1.71, 2.53)	< 0.01
2020-6	1 [Reference]		1 [Reference]	
2020-7	3.56 (2.85, 4.44)	< 0.01	2.15 (1.68, 2.76)	< 0.01
2020-8	2.48 (1.82, 3.37)	< 0.01	1.74 (1.24, 2.44)	< 0.01
2020-9	3.12 (2.33, 4.17)	< 0.01	1.71 (1.24, 2.36)	< 0.01
2020-10 (delta)	4.39 (3.65, 5.29)	< 0.01	2.15 (1.73, 2.68)	< 0.01
2020-11	5.40 (4.56, 6.40)	< 0.01	2.36 (1.91, 2.90)	< 0.01
2020-12 (beta)	5.55 (4.75, 6.49)	< 0.01	2.18 (1.78, 2.68)	< 0.01
2021-1 (gamma)	6.4 (5.30, 7.73)	< 0.01	2.03 (1.59, 2.58)	< 0.01
2021-2 (theta)	2.95 (1.99, 4.36)	< 0.01	2.04 (1.32, 3.15)	< 0.01
2021-3	1.86 (1.00, 3.46)	0.05	1.47 (0.72, 3.00)	0.29
Hospital and outpatient				
#H admits 1 year	1.12 (1.08, 1.16)	< 0.01	1.02 (0.97, 1.08)	0.44
#SCU admits 1 year	1.21 (0.94, 1.55)	0.13	1.15 (0.83, 1.60)	0.41
#DAD proc 1 year	1.04 (1.01, 1.07)	0.01	1.01 (0.97, 1.06)	0.59
#NACRS proc 1 year	1.00 (0.99, 1)	0.47	1.00 (1.00, 1.00)	0.52
Comorbidities				
Num of Elixhauser	1.05 (1.04, 1.07)	< 0.01	1.03 (1.01, 1.05)	< 0.01
Fluid and electrolyte disorders	1.47 (1.35, 1.61)	< 0.01	1.19 (1.07, 1.34)	< 0.01
Diabetes	1.12 (1.03, 1.23)	0.01	1.11 (1.00, 1.23)	0.06
Metastatic cancer	1.92 (1.48, 2.5)	0.00	1.58 (1.14, 2.19)	< 0.01
Liver disease	1.24 (0.98, 1.56)	0.07	1.51 (1.15, 1.99)	< 0.01

*LTC* long-term care, *DSL* designated support living, *ED* emergency department, *H* hospital, *Y* year, *Proc* procedures.

We also investigated the adjusted associations between clinical risk factors and 60-day all-cause mortality, stratified by sex illustrated in Table 3. Despite the overall similarity in the results for both genders, a slightly stronger association was observed in females compared to males for being symptomatic. Females had an aORs of 2.10 (95% CI 1.76, 2.51, p < 0.01) for being symptomatic, while males had an aORs of 1.77 (95% CI 1.43, 2.19, p < 0.01). However, the aORs for a positive result of the Covid-19 test and 60-day all-cause mortality were higher in the male population (aORs 4.25, 95% CI 3.40, 5.32, p < 0.01) compared to the female population (aORs 3.26, 95% CI 2.72, 3.91, p < 0.01). For diabetes, the male population showed a significant association (aORs 1.22, 95% CI 1.04, 1.43, p = 0.01), while no significant association was found in females (aORs 0.99, 95% CI 0.85, 1.15, p = 0.91).

**Table 3:** Associations between clinical risk factors and 60-day cause-specific mortality in continuing care residents in Alberta who were confirmed tested first positive or first negative with a Covid-19 infection between, March 1, 2020, to March 31, 2021.

Characteristic	Multivariable			
	Male		Female	
	AORs (95% CI)	p-value	AORs (95% CI)	p-value
Age	1.05 (1.04,1.06)	< 0.01	1.05 (1.04,1.05)	< 0.01
Specimen collection location				
LTC	1 [Reference]		1 [Reference]	
DSL	1.04 (0.65, 1.64)	0.88	0.92 (0.62, 1.36)	0.690
ED	4.07 (2.82, 5.86)	< 0.01	4.38 (3.15, 6.10)	< 0.01
H	3.21 (2.15, 4.79)	< 0.01	3.78 (2.64, 5.42)	< 0.01
Resident during collection				
LTC	1 [Reference]		1 [Reference]	
DSL	0.56 (0.37, 0.86)	< 0.01	0.53 (0.37, 0.76)	< 0.01
Symptomatic during collection				
No	1 [Reference]		1 [Reference]	
Yes	1.77 (1.43, 2.19)	< 0.01	2.10 (1.76, 2.51)	< 0.01
Unknown	1.28 (1.05, 1.57)	0.02	1.47 (1.24, 174)	< 0.01
Result of the covid test				
Negative	1 [Reference]		1 [Reference]	
Positive	4.25 (3.40, 5.32)	< 0.01	3.26 (2.72, 3.91)	< 0.01
Specimen year-month collection				
2020-3 (original)	4.85 (3.48, 6.75)	< 0.01	4.50 (3.36, 6.01)	< 0.01
2020-4 (alpha)	3.16 (2.45, 4.08)	< 0.01	2.80 (2.27, 3.46)	< 0.01
2020-5	2.59 (1.92, 3.50)	< 0.01	1.92 (1.48, 2.48)	< 0.01
2020-6	1 [Reference]		1 [Reference]	
2020-7	1.79 (1.16, 2.76)	< 0.01	2.50 (1.84, 3.40)	< 0.01
2020-8	1.76 (1.04, 2.99)	0.03	1.74 (1.12, 2.72)	0.01
2020-9	1.25 (0.71, 2.20)	0.43	2.19 (1.48, 3.24)	< 0.01
2020-10 (delta)	1.97 (1.39, 2.79)	< 0.01	2.29 (1.72, 3.05)	< 0.01
2020-11	2.61 (1.89, 3.61)	< 0.01	2.11 (1.60, 2.77)	< 0.01
2020-12 (beta)	2.45 (1.78, 3.38)	< 0.01	2.03 (1.55, 2.66)	< 0.01
2021-1 (gamma)	1.86 (1.26, 2.73)	< 0.01	2.32 (1.71, 3.16)	< 0.01
2021-2 (theta)	1.85 (0.93, 3.68)	0.08	2.30 (1.32, 3.99)	< 0.01
2021-3	0.80 (0.18, 3.49)	0.76	1.89 (0.80, 4.48)	0.15
Hospital and outpatient				
#H admits 1 year	1.00 (0.92, 1.08)	0.92	1.02 (0.94, 1.10)	0.67
#SCU admits 1 year	1.07 (0.67, 1.72)	0.77	1.02 (0.74, 1.76)	0.55
#DAD proc 1 year	1.00 (0.95, 1.06)	0.94	1.02 (0.95, 1.09)	0.56
#NACRS proc 1 year	1.00 (1.00, 1.00)	0.50	1.00 (1.00, 1.00)	0.59
Comorbidities				
Fluid and electrolyte disorders	1.12 (0.94, 1.34)	0.2	1.21 (1.04, 1.41)	0.01
Diabetes	1.22 (1.04, 1.43)	0.01	0.99 (0.85, 1.15)	0.91
Metastatic cancer	1.74 (1.09, 2.78)	0.02	1.4 (0.88, 2.24)	0.15
Liver disease	1.63 (1.09, 2.43)	0.02	1.31 (0.89, 1.93)	0.17

*LTC* long-term care, *DSL* designated support living, *ED* emergency department, *H* hospital, *Y* year, *Proc* procedures.

### Model performance

The univariable comparison in Appendix 10 shows that the ORs for the individual features of the initial data (without balancing) and data with balanced classes (using ROTE and SMOTE) were almost the same. After examination of the standard LR model (model 1–4), the critical weakness of predictive analytics was found to be the cut-off point^66^. After identifying the ideal sensitivity–specificity cut-off point using Youden’s index, we could improve the performance. The sensitivity (from 6 to 77%) and AUC (from 53 to 71%) scores for the LR model were increased by utilizing the optimal cut-point value (Table 4). Model 4 (LR model along with PT technique^67^, and ROTE methods) could achieve better performance in terms of sensitivity (1% increased) than model 2 (LR model without using pre-processing techniques).

**Table 4:** The performance metric of all the predictive modeling.

Machine learning models	Sensitivity (%)	AUC (%)	Cut-off point
Model 1 (LR model + initial data)	6	53	0.50
Model 2 (LR model + initial data + J)	77	71	0.083
Model 3 (LR model + PT + SMOTE + J)	75	72	0.46
Model 4 (LR model + PT + ROTE + J)	78	71	0.42
RF (PT + ROTE)	66	71	0.50
SVM (PT + ROTE)	73	72	0.50
XGBoost (PT + ROTE)	74	73	0.50
ANN (1-layer + PT + ROTE)	78	73	0.50
ANN (2-layer + PT + ROTE)	81	73	0.50
ANN (3-layer + PT + ROTE)	**82**	**73**	0.50

Significant values are in bold.

*LR* logistic regression, *PT* power transformation for normalizing the data, *SMOTE* synthetic minority over-sampling technique, *ROTE* random over-sampling technique, *J* Youden index, *RF* random forest, *SVM* support vector machine, *ANN* artificial neural network.

In this examination, by using optimal cut-off points and pre-processing methods the sensitivity (from 6 to 78%) and AUC (from 53 to 71%) scores were improved for the LR model (Table 4). Also, in this study, we examined three different ML models, including RF, SVM, XGBoost, and ANN. Among all, a 3-hidden-layers ANN model (included in Appendix 11) could accomplish the best performance in terms of sensitivity and AUC which was 82%, and 73% respectively. In Appendix 12, we present informative horizontal bar charts showcasing the sensitivity and AUC values of all the discussed models used in our study.

## Discussion

The primary outcome variable in this study was mortality within 60 days of a resident's first COVID-19 test. We aimed to identify factors associated with mortality among continuing care residents in Alberta during the COVID-19 pandemic and develop machine learning models to improve risk prediction for mortality. In this study, we employed a diverse range of databases, including EDW, ACCIS, NACRS, Claims, DAD, and AVS.

We identified several characteristics associated with mortality, including advanced age, male sex, metastatic cancer, chronic liver disease, having symptoms, previous SCU, and hospital admissions. Combining these factors achieved higher estimation in this population than if they were considered individually. Our findings were aligned with previous research as increased age, male sex, previous intensive care unit (ICU) admissions, and chronic conditions, like cancer and chronic liver disease, have been already recognized as risk factors for mortality in ill patients with COVID-19^9,68–71^.

The outcome variable of a study conducted by Panagiotou et al.^9^ was death due to any cause within 30 days of a resident's first positive COVID-19 polymerase chain reaction test result. The study leveraged unique electronic medical record data and other clinical data from a large multistate sample of US nursing homes. The study found that increased age, male sex, impaired cognitive and physical function, diabetes, chronic kidney disease, fever, shortness of breath, tachycardia, and hypoxia were independently associated with mortality in US nursing home residents with COVID-19. Understanding these risk factors can aid in the development of clinical prediction models of mortality in this population. Both our study and Panagiotou et al.^9^ identified increased age and male sex as risk factors for mortality in COVID-19 patients. Both studies also highlighted previous ICU admissions and chronic conditions (e.g., cancer and chronic liver disease) as risk factors. However, Panagiotou et al.^9^ focused on US nursing home residents, while our study may have a broader population.

The primary outcome variable was 28-day in-hospital mortality in a study done by Gupta et al.^68^. Other outcome variables included discharge from the hospital and remaining hospitalized at the end of the study follow-up. The study assessed 2215 adults with laboratory-confirmed COVID-19 who were admitted to intensive care units (ICUs) at 65 hospitals across the US from March 4 to April 4, 2020. The study identified demographic, clinical, and hospital-level risk factors that may be associated with death in critically ill patients with COVID-19. Factors independently associated with death included older age, male sex, higher body mass index, coronary artery disease, active cancer, and the presence of hypoxemia, liver dysfunction, and kidney dysfunction at ICU admission. Patients admitted to hospitals with fewer ICU beds had a higher risk of death. Hospitals varied considerably in the risk-adjusted proportion of patients who died and in the percentage of patients who received hydroxychloroquine, tocilizumab, and other treatments and supportive therapies. Our study and Gupta et al.^68^, both found older age and male sex to be risk factors for mortality in critically ill COVID-19 patients. But Gupta et al.^68^ focused on ICU-admitted patients and identified additional risk factors, including higher body mass index, coronary artery disease, active cancer, and specific organ dysfunctions.

The outcome variables of the study performed by Kuderer et al.^70^ were severe COVID-19 illness, hospitalization, admission to the ICU, mechanical ventilation, and death. The data sources were electronic medical records and patient-reported outcomes. The key findings of the study were that patients with cancer who contracted COVID-19 had a higher risk of severe illness, hospitalization, admission to the ICU, mechanical ventilation, and death compared to the general population. Additionally, patients with active cancer and those receiving cancer treatment had a higher risk of these outcomes compared to those with a history of cancer or those not receiving treatment. Our study, similar to Kuderer et al.^70^ revealed that cancer patients with COVID-19 faced an elevated risk of death compared to the general population. Yet, Kuderer et al.^70^ concentrated on COVID-19 outcomes in cancer patients, whereas our study encompassed a more diverse patient population.

Williamson et al.^71^ examined factors associated with COVID-19-related death. The study analyzed primary care records of 17,278,392 adults linked to 10,926 COVID-19-related deaths. Key findings showed associations between COVID-19-related death and male gender, greater age, deprivation, diabetes, severe asthma, and other medical conditions. Black and South Asian individuals had a higher risk, even after adjusting for other factors. The study provided valuable insights from one of the largest cohort studies on this topic. Our study, like Williamson et al.^71^ observed associations between COVID-19-related death and male gender, greater age, and specific medical conditions such as diabetes and severe asthma. However, while Williamson et al.^71^ analyzed primary care records, our study potentially utilized different data sources.

Grasselli et al.^69^, evaluated independent risk factors associated with mortality of COVID-19 patients treated in ICUs in Lombardy, Italy. The study included 3988 critically ill patients with laboratory-confirmed COVID-19 referred for ICU admission from February 20 to April 22, 2020. Key findings revealed that older age, male sex, high fraction of inspired oxygen, high positive end-expiratory pressure or low Pao2:Fio2 ratio on ICU admission, and history of chronic obstructive pulmonary disease, hypercholesterolemia, and type 2 diabetes were independently associated with mortality. Our study, along with Grasselli et al.^69^ identified older age and male sex as risk factors for mortality in COVID-19 patients. However, Grasselli et al.^69^ focused on patients treated in ICUs, whereas our study may have a broader population.

We also conducted gender-specific analyses to investigate the adjusted associations between clinical risk factors and 60-day all-cause mortality. While the overall results showed similarities between both genders, a slightly stronger association was observed in females compared to males for being symptomatic. Notably, aORs for a positive result of the Covid-19 test and 60-day all-cause mortality were higher in males compared to females. Additionally, while diabetes was significantly associated with mortality in males, no significant association was found in females. These findings emphasize the importance of gender-specific analyses to better understand the impact of these clinical risk factors on mortality outcomes.

The finding in this study was that the mortality rates were higher among LTC residents with COVID-19 compared with DSL residents even though DSL and LTC residents are almost the same in terms of characteristics, such as age, sex, and comorbidities. Our study is the first in our jurisdiction to compare rates of mortality between LTC and DSL using administrative data. We discovered the importance of the cut-off point for predictive modeling using the Youden index improved the sensitivity and AUC which is aligned with previous studies^72^. In the investigation of the pre-processing methods using administrative data and the LR model (to identify the individuals at risk of 60-day mortality), we found that using normalization (power transformation)^73^ and balancing classes (over-sampling techniques)^74^, improved the sensitivity and AUC that is aligned with previous studies^17,47,51,52,75^. According to these studies, the large degree of the imbalanced classes (in our dataset the rate of the “survivor” class outweighed the rate of “death” class 9 to 1) lowered the sensitivity, and therefore strategies to improve the model should be considered^17^. In most of these studies, over-sampling techniques have been proposed to solve the imbalanced class issue.

In this study, the ANN model received the best performance in terms of sensitivity and AUC which aligns with previous studies. In a study done by Sanderson et al.^15^, they discussed that the ANN model could outperform the LR in terms of both sensitivities by 4% and AUC by 2%. In this work, the author stated that ANN models can learn the non-linear relationships between predictors and outcomes. Also, they are capable of scaling well to large datasets. Furthermore, the ANN model was advocated by other studies as well^59–64^. In our study, the ANN model outperformed the standard LR (model 4) in both sensitivity and AUC metrics by 4% and 2%, respectively. ANN may not offer a significant improvement over LR. In many cases, LR's advantage of providing clearer interpretations and similar performance makes it the preferred choice. However, in scenarios where enhanced sensitivity is needed, such as with large datasets or specific organizational requirements, ANN may be a more suitable option.

We had several limitations that should be acknowledged. First, the administrative data used was not collected for this specific purpose. However, by using existing administrative data, we were able to obtain results that would have otherwise been restricted by the cost of primary data collection^76^. By using secondary data sources, certain variables were restricted from use or pre-defined as part of the Alberta COVID-19 Analytics and Research Database. For example, some variables were already coded, and data linkage had been done already. The residual and unmeasured confounding in terms of facility-level and patient-level characteristics limited us from further exploration into the difference in the risk of death between LTC and DSL residents.

Second, the cohort was defined based on continuing care residents being tested for COVID-19, which means that those not tested for COVID-19 were not included potentially introducing a selection bias. However, we believe this was somewhat mitigated as we have captured most residents in continuing care based on previous estimates of the number of residents in continuing care^77^. Also, we had to rely on all-cause mortality data as we did not have access to the specific data required for calculating cause-specific mortality^78^. The vital statistics data lacked cause of death details, preventing us from categorizing deaths as COVID-related or not. Although we acknowledge that cause-specific mortality would provide a more comprehensive understanding, we believe that using all-cause mortality can still be a suitable proxy for deaths related to or indirectly caused by COVID-19.

Third, based on COVID-19 variants in Canada^79^, the Delta and Beta variants of Covid-19 emerged one month before and one month after November 2020, respectively. When considering the year and month of the specimen collection in our cohort, the odds of death in November 2020 and in December 2020 were highest. Yet, we did not have enough data to know in which month the patients died.

Lastly, the Youden index, which was used in this study is a measure of the overall performance of a model and is not directly related to calibration. We opted to use the cut-off point rather than calibration due to its simplicity and ease of interpretation. Calibration, on the other hand, involves fine-tuning the model probabilities to match the observed outcomes, which can be more complex and computationally demanding. Investigating calibration in different risk strata would provide additional information about the performance of the model. In expressions of ML algorithms, the interplay between overfitting and underfitting was a challenge as the success of these algorithms depends on the selection of the parameters according to the number of observations, and features^80^.

In conclusion, in this cohort study of Alberta continuing care residents tested for COVID-19, the all-cause 60-day mortality rate was 10.12%. COVID-19 test results and other characteristics, metastatic cancer, chronic liver disease, advancing age, and male sex, increased the risk of death. The LTC residents were also at higher risk of death compared to the DSL residents. We examined the pre-processing methods and ML models for predicting mortality and found that the combination of normalization, random oversampling techniques, and three-layer neural network classification provided superior prediction to other ML models. Our findings can enhance treatment decisions in healthcare. Further exploration is needed to harness the potential of ANN models for improved outcomes. ANNs offer data-driven insights, leading to precise diagnoses, optimized treatment plans, and better patient outcomes. Rigorous validation and collaboration between healthcare professionals and AI experts are crucial for safe and effective integration in clinical practice.

### Supplementary Information

Supplementary Information.

## Data Availability

The data for this study was provided by AHS. Due to the sensitivity of the information, AHS has restricted the use of the data and they are not publicly available. To obtain the data, researchers will need approval from a certified research ethics board in Alberta as well as a Data Sharing Agreement from AHS through ethics boards. The websites for ethics boards and data sharing agreements are respectively located at https://hreba.ca/ and https://www.albertahealthservices.ca/research/Page16074.aspx.
