# Supplementary material for: Machine learning risk estimation and prediction of death in continuing care facilities using administrative data
Source: Sci Rep. 2023 Oct 18;13:17708. doi: 10.1038/s41598-023-43943-9 (PMC10584843; doi:10.1038/s41598-023-43943-9)
Supplement: Supplementary file 1 — Supplementary Information. [file 41598_2023_43943_MOESM1_ESM.docx]

**APPENDIX 1:** The RECORD checklist of items that were reported in this study.

|  | **RECORD items** | **Location in manuscript** |
| --- | --- | --- |
| **Title and abstract** | |  |
|  | RECORD 1.1: The type of data used should be specified in the title or abstract. When possible, the name of the databases used should be included. | The database was stated in the title and abstract. |
|  | RECORD 1.2: If applicable, the geographic region and timeframe within which the study took place should be reported in the title or abstract. | The location was stated in the title and abstract. |
|  | RECORD 1.3: If linkage between databases were conducted for the study, this should be clearly stated in the title or abstract. | The linkage was stated in the abstract. The linkage clearly stated in the method. |
| **Methods** |  |  |
| **Participants** | RECORD 6.1: The methods of study population selection (such as codes or algorithms used to identify subjects) should be listed in detail. If this is not possible, an explanation should be provided. | In the method of the study, the population selection was explained. |
|  | RECORD 6.2: Any validation studies of the codes or algorithms used to select the population should be referenced. If validation was conducted for this study and not published elsewhere, detailed methods and results should be provided. | This is not applicable as our population was explained in RECORD 6.1. |
|  | RECORD 6.3: If the study involved linkage of databases, consider use of a flow diagram or other graphical display to demonstrate the data linkage process, including the number of individuals with linked data at each stage. | In Appendix 3, we illustrated all the steps to show the steps we took to create the cohort. |
| **Variables** | RECORD 7.1: A complete list of codes and algorithms used to classify exposures, outcomes, confounders, and effect modifiers should be provided. If these cannot be reported, an explanation should be provided. | All the codes were reported in Appendix 2. |
| **Data access and**  **cleaning methods** | RECORD 12.1: Authors should describe the extent to which the investigators had access to the database population used to create the study population. | All the data sources that we had access to were explained in the data source section of the method. |
|  | RECORD 12.2: Authors should provide information on the data cleaning methods used in the study. | Data cleaning was explained in the methodology, and the steps clearly defined in Appendix 3. |
| **Linkage** | RECORD 12.3: State whether the study included person-level, institutional-level, or other data linkage across two or more databases. The methods of linkage and methods of linkage quality evaluation should be provided. | Person-level linkage was performed, and it is clearly stated in the linkage part in the methodology and illustrated in Appendix 3. |
| **Results** |  |  |
| **Participants** | RECORD 13.1: Describe in detail the selection of the persons included in the study (i.e., study population selection) including filtering based on data quality, data availability and linkage. The selection of included persons can be described in the text and/or by means of the study flow diagram. | The selection of the persons all described in the results, and it was clearly illustrated in the flowchart in Appendix 3. |
| **Discussion** |  |  |
| **Limitations** | RECORD 19.1: Discuss the implications of using data that were not created or collected to answer the specific research question(s). Include discussion of misclassification bias, unmeasured confounding, missing data, and changing eligibility over time, as they pertain to the study being reported. | The limitation of the study is clearly stated in the discussion. |
| **Other Information** |  |  |
|  | RECORD 22.1: Authors should provide information on how to access any supplemental information such as the study protocol, raw data, or programming code. | supplemental information is submitted to the journal. Data access and programming codes are available in the Data Availability section. |

**APPENDIX 2:** Data sources and the Codes in Details:

| **Data Source** | **Codes** | **Details** |
| --- | --- | --- |
| 1. DAD | ICD-10-CA for comorbidities ^(32)^ | Type: DXCODE1 - DXCODE 25 |
|  | ICD-10-CA for ICU admission | Any SCU code other than 90, 93, 95, 98, or 99 |
|  | ICD-10-CA for influenza-like illness | B97.89, H66.9, J00, J01, J06, J09, J10, J11, J12, J18, J20, J21, J22, R05, R50.9 |
|  | ICD-10-CA for CV-ICU admission | SCU codes equal to 40 or 45 |
|  | Ventilation admission | CCI code equal to 1. GZ.31 |
|  | CCI Procedure codes | PROCCODE1 - PROCCODE 20 |
| 2- AHCIP | ICD9 for comorbidities | HLTH_DX_ICD9x_CODE_1  HLTH_DX_ICD9x_CODE_2  HLTH_DX_ICD9x_CODE_3 |

**APPENDIX 3:** The diagram flowchart of the study as described in the methodology. Abbreviations: LTC, long-term care; DSL, designated support living; ULIs, universal lifetime identifier; PHN, personal health number; DAD, discharge abstract database; NACRS, national ambulatory care reporting system.

**APPENDIX 4:** The hyperparameters for each ML (machine learning) model.

| **Model** | **Optimum hyperparameters** | **Experimented hyperparameters** | **# Trials** |
| --- | --- | --- | --- |
| RF (random forest) | bootstrap= ‘TRUE’  max_depth= 10  max_features= ‘sqrt’  min_samples_leaf= 8  min_samples_split= 10  n_estimators= 600  criterion= 'entropy' | bootstrap= (‘True', 'False’)  max_depth= (10, 20, 30, 40, 50, 60, 70, 80, 90, 100)  max_features= (‘auto', 'sqrt', 'log2')  min_samples_leaf= (1, 2, 4, 8)  min_samples_split= (2, 5, 10)  n_estimators= (400, 600, 800, 1000, 1200, 1400, 1600, 1800, 2000)  criterion= (‘gini', 'entropy') | 12,960 |
| SVM (support vector machine) | C= 1000,  Gamma= 0. 0001,  Kernel= ‘sigmoid’ | C= (0.1, 1., 10., 100., 1000., 1200., 1300., 1400., 1500., 2000., 3000)  gamma= (0.000001, 0.00001, 0.0001, 0.001, 0.01, 0.1, 1.0 ,10.0, 100.0, 'scale', 'auto’)  kernel= (‘rbf' 'poly' 'sigmoid’) | 396 |
| XGBoost (eXtreme gradient boosting) | subsample=0.7  n_estimators=1000  max_depth=1000  learning_rate=0.01  colsample_bytree=0.5  colsample_bylevel=0.6 | subsample= (0.7, 0.8, 0.9)  n_estimators= (100, 500, 1000)  max_depth= (3, 5, 6, 10, 15, 20)  learning_rate= (0.01, 0.1, 0.2, 0.3, 0.4)  colsample_bytree= (0.4, 0.5, 0.6, 0.7, 0.8, 0.9)  colsample_bylevel= (0.4, 0.5, 0.6, 0.7, 0.8, 0.9) | 9,720 |
| ANN (artificial neural networks) (1 layer) (1 layer) | hidden_layer_sizes= (2,)  max_iter= 100  activation= ' logistic'  solver= 'lbfgs’  alpha= 0.001  learning_rate= 'adaptive' | hidden_layer_sizes= (1 - 32)  max_iter= (50, 100, 150, 1000)  activation= (‘tanh', 'relu', 'logistic')  solver= (‘sgd' 'adam' 'lbfgs’)  alpha= (1e-5, 0.00001, 0.0001, 0.001, 0.01, 0.05)  learning_rate= (‘constant', 'adaptive') | 13,824 |
| ANN (2 layers) | hidden_layer_sizes= (1, 14)  max_iter= 1000000  activation= 'relu'  solver= 'lbfgs’  alpha= 0.05  learning_rate= 'adaptive' | hidden_layer_sizes_1= (1 - 32)  hidden_layer_sizes_2= (1 - 32)  max_iter= (1000000)  activation= (‘tanh', 'relu', 'logistic')  solver= (‘sgd' 'adam' 'lbfgs’)  alpha= (1e-5, 0.00001, 0.0001, 0.001, 0.01, 0.05)  learning_rate= (‘constant', 'adaptive') | 110,592 |
| ANN (3 layers) | hidden_layer_sizes= (1, 21, 17)  max_iter= 1000000  activation= 'relu'  solver= 'lbfgs’  alpha= 1e-5  learning_rate= 'adaptive' | hidden_layer_sizes_1= (1 - 32)  hidden_layer_sizes_2= (1 - 32)  hidden_layer_sizes_3= (1 - 32)  max_iter= (1000000)  activation= ('relu')  solver= ('lbfgs’)  alpha= (1e-5)  learning_rate= ('adaptive') | 32,768 |

**APPENDIX 5:** Packages of the Python programming language used in this study.

| **# Number** | **Package** | **# Number** | **Package** |
| --- | --- | --- | --- |
| 1 | Pandas | 4 | Imblearn |
| 2 | Numpy | 5 | Os |
| 3 | Sklearn | 6 | Sys |

**APPENDIX 6:** The most prevalent comorbidities of continuing care residents in Alberta who were confirmed tested positive or negative with a Covid-19 infection between March 1, 2020, to March 31, 2021.

|  |  | **60-day mortality** | |  |
| --- | --- | --- | --- | --- |
| **Comorbidities** |  | **Yes=2590 (10.12%)** | **No=22990(89.87%)** | **Total=25580** |
| Num of Elixhauser category | 1+ | 2406 (92.9) | 21432 (93.22) | 23838 (93.19) |
|  | 0 | 184 (7.1) | 1558 (6.78) | 1558 (6.81) |
| Hypertension, uncomplicated |  | 1307 (50.46) | 11751 (51.11) | 13058 (51.05) |
| Depression |  | 1121 (43.28) | 10922 (47.51) | 12043(47.08) |
| Other neurological disorders |  | 1060 (40.93) | 9413 (40.94) | 10473 (40.94) |
| Diabetes |  | 802 (30.97) | 6557 (28.52) | 7359 (28.77) |
| Rheumatoid arthritis/ collagen vascular diseases |  | 699 (26.99) | 6457 (28.09) | 7156 (27.98) |
| Psychoses |  | 705 (27.22) | 5845 (25.42) | 6550 (25.61) |
| Fluid and electrolyte disorders |  | 809 (31.24) | 5413 (23.55) | 6222 (24.32) |
| Cardiac arrhythmias |  | 745 (28.76) | 5252 (22.84) | 5997 (23.44) |
| Chronic pulmonary disease |  | 658 (25.41) | 4702 (20.45) | 5360 (20.95) |
| Congestive heart failure |  | 726 (28.03) | 4508 (19.61) | 5234 (20.46) |
| Renal failure |  | 443 (17.1) | 3086 (13.42) | 3529 (13.80) |
| Liver disease |  | 84 (3.24) | 607 (2.64) | 691 (2.7) |
| Metastatic cancer |  | 69 (2.66) | 323 (1.4) | 392 (1.53) |

**APPENDIX 7:** The horizontal bar chart depicts the characteristics of continuing care residents in Alberta who died (2,590 residents, 10.12%) and were tested positive or negative for Covid-19 between March 1, 2020, and March 31, 2021. The x-axis represents the count, while the y-axis represents the clinical risk factors. Abbreviations: LTC, long-term care; DSL, designated support living; ED, emergency department; H, hospital.

**APPENDIX 8:** The horizontal bar chart illustrates the multivariable associations between clinical risk factors and 60-day all-cause mortality in continuing care residents in Alberta. The study includes residents who tested positive or negative for Covid-19 between March 1, 2020, and March 31, 2021. The chart displays the adjusted odds ratios (aORs) with their 95% confidence intervals (CI) and corresponding p-values for each risk factor. The x-axis represents the adjusted odds ratios (aORs) along with their corresponding 95% confidence intervals (CIs), while the y-axis represents the clinical risk factors. Abbreviations: LTC, long-term care; DSL, designated support living; ED, emergency department; H, hospital; Y, year.

**APPENDIX 9:** Associations between the most prevalent comorbidities (considered together with clinical, inpatient, and demographic characteristics) and 60-day all-cause mortality in continuing care residents in Alberta who were confirmed tested first positive or first negative with a Covid-19 infection between, March 1, 2020, to March 31, 2021.

|  |  | **Univariable** |  | **Multivariable** |  |
| --- | --- | --- | --- | --- | --- |
|  |  | **ORs (95% CI)** | **P-value** | **ORs (95% CI)** | **P-value** |
| **Comorbidities** |  |  |  |  |  |
| Num of Elixhauser |  | 1.05 (1.04, 1.07) | < 0.01 | 1.03 (1.01, 1.05) | < 0.01 |
| Hypertension, uncomplicated | Yes | 0.97 (0.9, 1.06) | 0.53 | 0.79 (0.71, 0.88) | < 0.01 |
| Depression | Yes | 0.84 (0.78, 0.91) | < 0.01 | 0.94 (0.86, 1.04) | 0.26 |
| Other neurological disorders | Yes | 1.00 (0.92, 1.08) | 0.99 | 0.96 (0.88, 1.06) | 0.46 |
| Diabetes | Yes | 1.12 (1.03, 1.23) | 0.01 | 1.11 (1.00, 1.23) | 0.06 |
| Rheumatoid arthritis/ collagen vascular diseases | Yes | 0.95 (0.86, 1.4) | 0.24 | 0.82(0.74, 0.92) | < 0.01 |
| Psychoses | Yes | 1.1 (1, 1.2) | 0.05 | 1.05 (0.94, 1.17) | 0.4 |
| Fluid and electrolyte disorders | Yes | 1.47 (1.35, 1.61) | < 0.01 | 1.19 (1.07, 1.34) | < 0.01 |
| Cardiac arrhythmias | Yes | 1.36 (1.25, 1.5) | < 0.01 | 1.04 (0.93, 1.17) | 0.51 |
| Chronic pulmonary disease | Yes | 1.32 (1.2, 1.45) | < 0.01 | 1.14 (1.02, 1.28) | 0.16 |
| Congestive heart failure | Yes | 1.6 (1.46, 1.75) | < 0.01 | 1.2 (1.06, 1.35) | < 0.01 |
| Renal failure | Yes | 1.33 (1.19, 1.48) | < 0.01 | 1.09 (0.95, 1.25) | 0.2 |
| Liver disease | Yes | 1.24 (0.98, 1.56) | 0.07 | 1.51 (1.15, 1.99) | < 0.01 |
| Metastatic cancer | Yes | 1.92 (1.48, 2.5) | 0.00 | 1.58 (1.14, 2.19) | < 0.01 |

**APPENDIX 10:** The comparison of the data with and without balancing class methods (ROTE and SMOTE) in terms of ORs (95% CI) and P-values by using a univariable logistic regression.

|  | **Initial data** | | **Balanced with ROTE** | | **Balanced with SMOTE** | |
| --- | --- | --- | --- | --- | --- | --- |
|  | **OR** | **P-value** | **OR** | **P-value** | **OR** | **P-value** |
| Age category |  |  |  |  |  |  |
| 80+ | 1[Reference] |  | 1[Reference] |  | 1[Reference] |  |
| 70-79 | 0.61 | 0.00 | 0.62 | 0.00 | 0.62 | 0.00 |
| 60-69 | 0.45 | 0.00 | 0.47 | 0.00 | 0.47 | 0.00 |
| 50-59 | 0.20 | 0.00 | 0.20 | 0.00 | 0.20 | 0.00 |
| 40-49 | 0.1 | 0.00 | 0.09 | 0.00 | 0.09 | 0.00 |
| 30-39 | 0.19 | 0.00 | 0.23 | 0.00 | 0.23 | 0.00 |
| 18-29 | 0.32 | 0.18 | 0.24 | 0.00 | 0.24 | 0.00 |
| Age (continuous) | 1.04 | 0.00 | 1.04 | 0.00 | 1.04 | 0.00 |
| Gender |  |  |  |  |  |  |
| Male | 1.40 | 0.00 | 1.41 | 0.00 | 1.40 | 0.00 |
| Female | 1[Reference] |  | 1[Reference] |  | 1[Reference] |  |
| Specimen collection location |  |  |  |  |  |  |
| Long-term care | 1[Reference] |  | 1[Reference] |  | 1[Reference] |  |
| Designated supported living | 0.42 | 0.00 | 0.43 | 0.00 | 0.43 | 0.00 |
| Emergency department | 3.09 | 0.00 | 3.09 | 0.00 | 3.27 | 0.00 |
| Hospital | 2.33 | 0.00 | 2.51 | 0.00 | 2.4 | 0.00 |
| Resident during collection |  |  |  |  |  |  |
| Long term care | 1[Reference] |  | 1[Reference] |  | 1[Reference] |  |
| Designated supported living | 0.49 | 0.00 | 0.5 | 0.00 | 0.51 | 0.00 |
| Symptomatic during collection |  |  |  |  |  |  |
| No | 1[Reference] |  | 1[Reference] |  | 1[Reference] |  |
| Yes | 2.33 | 0.00 | 2.37 | 0.00 | 2.37 | 0.00 |
| Unknown | 2.02 | 0.00 | 1.99 | 0.00 | 1.99 | 0.00 |
| Result of the covid test |  |  |  |  |  |  |
| Negative | 1[Reference] |  | 1[Reference] |  | 1[Reference] |  |
| Positive | 4.25 | 0.00 | 4.24 | 0.00 | 4.17 | 0.00 |
| Specimen year-month collection |  |  |  |  |  |  |
| 2020-3 (Original variant) | 6.25 | 0.00 | 6.42 | 0.00 | 6.42 | 0.00 |
| 2020-4 (Alpha) | 4.01 | 0.00 | 4.13 | 0.00 | 4.13 | 0.00 |
| 2020-5 | 3.59 | 0.00 | 3.69 | 0.00 | 3.69 | 0.00 |
| 2020-6 | 1[Reference] |  | 1[Reference] |  | 1[Reference] |  |
| 2020-7 | 3.56 | 0.00 | 3.51 | 0.00 | 3.51 | 0.00 |
| 2020-8 | 2.48 | 0.00 | 2.34 | 0.00 | 2.34 | 0.00 |
| 2020-9 | 3.12 | 0.00 | 3.18 | 0.00 | 3.18 | 0.00 |
| 2020-10 (Delta) | 4.39 | 0.00 | 4.41 | 0.00 | 4.41 | 0.00 |
| 2020-11 | 5.40 | 0.00 | 5.64 | 0.00 | 5.64 | 0.00 |
| 2020-12 (Beta) | 5.55 | 0.00 | 5.77 | 0.00 | 5.77 | 0.00 |
| 2021-1 (Gamma) | 6.4 | 0.00 | 6.22 | 0.00 | 6.22 | 0.00 |
| 2021-2 (Theta) | 2.945 | 0.00 | 3.27 | 0.00 | 3.27 | 0.00 |
| 2021-3 | 1.86 | 0.05 | 1.94 | 0.00 | 1.94 | 0.00 |
| Year-month collection continuous | 1.08 | 0.00 | 1.07 | 0.00 | 1.07 | 0.00 |
| Num of Elixhauser category |  |  |  |  |  |  |
| 1+ | 1[Reference] |  | 1[Reference] |  | 1[Reference] |  |
| 0 | 1.05 | 0.53/0.53 | 1.07 | 0.05 | 1.04 | 0.29 |
| Elixhauser features for 2 years |  |  |  |  |  |  |
| Elixhauser index | 1.03 | 0.00 | 1.03 | 0.00 | 1.03 | 0.00 |
| Num of Elixhauser | 1.05 | 0.00 | 1.05 | 0.00 | 1.05 | 0.00 |
| Num of admits for 1 year |  |  |  |  |  |  |
| Hospital | 1.12 | 0.00 | 1.12 | 0.00 | 1.12 | 0.00 |
| SCU | 1.21 | 0.13 | 1.22 | 0.00/0.00 | 1.24 | 0.00 |
| Num procedures for 1 year |  |  |  |  |  |  |
| DAD | 1.04 | 0.01 | 1.04 | 0.00 | 1.04 | 0.00 |
| NACRS | 1.00 | 0.47 | 1.00 | 0.55 | 1.00 | 0.04 |
| Comorbidities |  |  |  |  |  |  |
| Hypertension, uncomplicated | 0.97 | 0.53 | 1.0 | 0.82 | 0.97 | 0.13 |
| Depression | 0.84 | 0.00 | 0.84 | 0.00 | 0.83 | 0.00 |
| Other neurological disorders | 1.00 | 0.99 | 0.99 | 0.58 | 0.99 | 0.75 |
| Diabetes | 1.12 | 0.01 | 1.12 | 0.00 | 1.13 | 0.00 |
| Rheumatoid arthritis/ collagen vascular diseases | 0.95 | 0.24 | 0.95 | 0.00 | 0.95 | 0.01 |
| Psychoses | 1.1 | 0.05 | 1.08 | 0.00 | 1.07 | 0.00 |
| Fluid and electrolyte disorders | 1.47 | 0.00 | 1.48 | 0.00 | 1.49 | 0.00 |
| Cardiac arrhythmias | 1.36 | 0.00 | 1.36 | 0.00 | 1.36 | 0.00 |
| Chronic pulmonary disease | 1.32 | 0.00 | 1.33 | 0.00 | 1.32 | 0.00 |
| Congestive heart failure | 1.6 | 0.00 | 1.62 | 0.00 | 1.62 | 0.00 |
| Renal failure | 1.33 | 0.00 | 1.30 | 0.00 | 1.32 | 0.00 |
| Liver disease | 1.24 | 0.07 | 1.15 | 0.01 | 1.23 | 0.01 |
| Metastatic cancer | 1.92 | 0.00 | 1.91 | 0.00 | 1.87 | 0.00 |

**APPENDIX 11:** The architecture and the results for the ANN model.

**APPENDIX 12:** The horizontal bar chart illustrates the sensitivity and the area under the curve (AUC) values of different machine learning models employed in the study. The models are sorted based on their performance in both visualizations. Remarkably, the ANN with three layers demonstrated the highest performance in terms of both sensitivity and AUC. Abbreviations: LR, logistic regression; PT, power transformation for normalizing the data; SMOTE, synthetic minority over-sampling technique; ROTE, random over-sampling technique; J, Youden index; RF, random forest; SVM, support vector machine; ANN, artificial neural network.
